# Overexpression of carboxypeptidase X M14 family member 2 predicts an unfavorable prognosis and promotes proliferation and migration of osteosarcoma

**DOI:** 10.1186/s13000-019-0887-0

**Published:** 2019-10-24

**Authors:** Xin Zhao, Ronghang Li, Qian Wang, Minfei Wu, Yanbing Wang

**Affiliations:** 1 Orthopedic Department, The Second Hospital of Jilin University, No. 128 Ziqiang Road, Changchun, 130041 China; 2 Department of Joint Surgery and Sports Medicine, The Second Hospital of Jilin University, No. 128 Ziqiang Road, Changchun, 130041 China; 3 Otolaryngology Head and Neck Surgery, First Hospital of Jilin University, Changchun, Jilin 130021 People’s Republic of China

**Keywords:** Carboxypeptidase X, M14 family member 2, Epithelial mesenchymal transition, Metastasis, Osteosarcoma

## Abstract

**Background:**

Carboxypeptidase X, M14 family member 2 (CPXM2), has been associated with several human developmental disorders. However, whether CPXM2 is involved in oncogenesis or tumor progression remains unclear. Currently, the clinical relevance and function of CPXM2 in human osteosarcoma were investigated.

**Materials and methods:**

The expression of CPXM2 in osteosarcoma cell lines and tissues were explored by immunohistochemistry and western blotting assays. A eukaryotic expression plasmid was transfected into fetal osteoblast cells to overexpress CPXM2 and the endogenous CPXM2 in osteosarcoma cells was silenced through an RNA interference (RNAi) method transfection. These transfections were validated via western blotting, and the expression levels of several key molecules involved in the epithelial mesenchymal transition was also determined via western blotting. The expression levels of CPXM2 in a fetal osteoblast cell line with CPXM2 overexpressing and an osteosarcoma CPXM2-knockout cell line was confirmed via reverse transcription-quantitative polymerase chain reaction (RT-qPCR), western blotting and immunofluorescence. The malignant phenotype of osteosarcoma cells was indicated by the cholecystokinin octapeptide, colony formation assay, scratch wound healing assay, and Transwell® migration assay.

**Results:**

We found that CPXM2 was overexpressed in osteosarcoma and that the overexpression was associated with an unfavorable prognosis and tumor node metastasis staging. The knockdown of CPXM2 in cultured osteosarcoma cells significantly impeded cell proliferation and migration. In addition, the upregulation of CPXM2 in fetal osteoblast cells significantly promoted cell proliferation and migration. Besides, western blotting results revealed that several key molecules involved in the epithelial mesenchymal transition (EMT) were regulated by CPXM2.

**Conclusion:**

Taken together, these results imply an active role for CPXM2 in promoting tumor aggressiveness via epithelial to mesenchymal transition (EMT) modulation in osteosarcoma.

## Background

Generally, proteins undergo post-translational modifications (PTMs) to impact the functions of the protein [1] . Proteolysis, a well-known PTM comprises more than 500 identified proteases and peptidases which can modulate or active the activity of proteins and peptides [2]. Release of C-terminal amino acids are a widespread process that plays a role in degradation, processing, and modulation of proteins and peptides [3]. Carboxypeptidases (CPs) were revealed to conduct numerous varied physiological functions via eliminating C-terminal amino acids from peptides and proteins [4]. For instance, carboxypeptidase D is the main contributor for antibody C-terminal lysine cleavage in Chinese hamster ovary cells [5]. Carboxypeptidase E (CPE) is a novel regulator of the canonical Wnt signaling pathway and have a potential role in cell proliferation, cell fate determination and tumorigenesis [6]. The genetic defects in CPs have been involved with numerous developmental diseases [7]. A recent study revealed that Carboxypeptidase E is essential for migration and dendrite arborization of cortical neuron.

Although not all are recognized to be active as peptidases, the M14 family proteins including 25 distinct family members of carboxypeptidase is the largest family of enzymes responsible for cleavage of C-terminal residues in most mammals [8, 9]. The M14 family members performed diverse biological impacts owing to their variable tissue, cellular, and subcellular distributions and substrate specificities [5, 10]. Carboxypeptidase X, M14 family member 2 (CPXM2) have been reported to be involved in cell-cell interactions and associated with developmental diseases [11, 12], late-onset Alzheimer’s disease, and cognitive decline in schizophrenia [13, 14]. However, whether CPXM2 is involved in oncogenesis or tumor progression remains unclear.

The main reason for the poor prognosis of osteosarcoma is metastasis and recurrence, and the overall 5-year survival rate is less than 20% [15, 16]. To date, restraining the recurrence and metastasis of osteosarcoma has been shown to be limited in the therapy of this disease, and once tumors progress into the metastatic stage, there has been no feasible efficient therapy until now [17]. Hence, it is of vital importance to elucidate the molecular mechanisms that lead to disease progression of osteosarcoma. In the current study, we found that CPXM2 was overexpressed in osteosarcoma compared to normal bone tissues. However, at present there are no reports documenting the impact of CPXM2 in osteosarcoma tumorigenesis.

## Materials and methods

### Antibodies

Rabbit anti-human CPXM2 (cat. no. ab201077) and mouse anti-human β-actin (cat. no. ab8226) were purchased from Abcam (Cambridge, MA, USA). Rabbit monoclonal anti-human E-Cadherin (cat. no. 3195), N-Cadherin (cat. no. 13116), Vimentin (cat. no. 5741) and ZEB1 (cat. no. 3336) were purchased from Cell Signaling Technology, Inc. (Danvers, MA, USA). Horseradish peroxidase-conjugated secondary antibody (cat. no. sc-2357) were purchased from Santa Cruz Biotechnology, Inc., Dallas, TX, USA.

### Cell culture and transfection

Human osteosarcoma cell lines (Saos2, 143B, MG63, and U2OS) and a human fetal osteoblast cell line (hFOB.1.19) were purchased from the Institute of Basic Medical Sciences, Chinese Academy of Medical Sciences (Beijing, China). The full-length fragments of CPXM2 were inserted into a pNSE-IRES2-EGFP-C1 vector to generate a CPXM2 overexpression plasmid (constructed and amplified by KeyGEN BioTECH), with empty pNSE-IRES2-EGFP-C1 vectors serving as a negative control (vector). hFOB.1.19 cells were seeded into 6-well plates at a density of 1.2 × 105 cells/well, followed by transfected with the above products with Lipofectamine 2000 reagent (Invitrogen; Thermo Fisher Scientific, Inc.), and G418 (Sigma-Aldrich, Merck KGaA, Darmstadt, Germany) was used to expand G418-resistant clones in culture as a monoclonal population.

### RNA extraction and the quantitative polymerase chain reaction (qPCR)

Total RNA was isolated from cell cultures or from snap-frozen tissues from osteosarcoma patients using RNAiso Plus (Takara Bio, Kusatsu, Japan) according to the manufacturer’s instructions, reverse transcribed with HiScript Q Select RT SuperMix (R132–01, Vazyme, Jiangsu, China) according to the manufacturer’s protocol, and subjected to real-time reverse transcription (RT)-PCR using the 2^-ΔΔCT^ method [18]. The thermocycling conditions were as follows: 95 °C for 30 s, followed by 40 cycles of 95 °C for 10 s, 60 °C for 32 s, 95 °C for 15 s, 60 °C for 60 s and 95 °C for 15 s. Each sample was determined in duplicate. All PCR products were confirmed by 2.0% agarose gel electrophoresis. The sequences for RT-PCR primers were: CPXM2 forward primer, 5′-GTGCGCGGGAAGAAATGAC-3′; and reverse primer, 5′-CCTCCCTTGAGTGATGACACC-3′. The specificity of primers was validated by sequencing. Glyceraldehyde 3-phosphate dehydrogenase (GAPDH) served as an internal control. Experiments were repeated three times in duplicate.

### Western blotting

Total protein was extracted from cell cultures or homogenized tissues from osteosarcoma patients using radioimmunoprecipitation assay lysis buffer (Beyotime Biotechnology, Shanghai, China) containing phenylmethylsulfonyl fluoride (Beyotime Biotechnology) and proteinase inhibitor cocktail solution (Roche, Basel, Switzerland), and quantitated using the bicinchoninic acid protein assay (Beyotime Biotechnology) as recommended by the manufacturers. Western blotting was performed according to standard methods as previously described [18]. Briefly, Proteins (30 μg) were separated by 10% SDS-PAGE and then transferred to a polyvinylidene fluoride (PVDF) membrane. Subsequently, the membranes were blocked with 5% fat-free dry milk at room temperature for 1 h. Then, the blots were incubated with a rabbit anti-CPXM2 and β-actin antibody (1:1000 dilution; Abcam, ambridge, MA, USA), and rabbit monoclonal antibodies against E-Cadherin, N-Cadherin, Vimentin and ZEB1 (1:1000 dilution; Cell Signaling Technology, Danvers, MA, USA) at 4 °C overnight. The membranes were again washed with TBST and incubated with respective horseradish peroxidase (HRP)-conjugated secondary antibodies (1:2000; goat anti-rat cat. no. A0192, goat anti-mouse cat. no. A0216 and goat anti-rabbit cat. no. A0239, Beyotime, Jiangsu, China) at room temperature for 1 h. The proteins were finally examined by an enhanced chemiluminescence system (ECL) (P0018AS, Beyotime, Jiangsu, China). The grayscale values of protein bands were analyzed using ImageJ software (National Institutes of Health, Bethesda, MD, USA).

### Cell proliferation assay

hFOB.1.19 or U2OS cells were seeded at a density of 3 × 10^5^ cells/well into 36-well plates in triplicate and cultured at 37 °C overnight in an incubator. A growth curve was drawn based on the growth every 12 h over 4 days as analyzed via a colorimetric water-soluble tetrazolium salt kit (CCK-8; Dojindo Molecular Technologies, Inc., Kumamoto, Japan) in accordance with the manufacturer’s protocol.

### Cell colony formation assay

A plate colony formation assay was performed as previously described [19]. Briefly, stably transfected hFOB.1.19 or U2OS cells (5 × 10^2^ cells/well) were seeded in six-well plates and cultivated in F12K or RPMI-1640 complete medium at 37 °C for 14 days. The cell colonies were washed with phosphate-buffered saline (PBS) twice, fixed with methanol for 20 min, and stained with 0.1% crystal violet in PBS (Beyotime Biotechnology) for 15 min. The colonies containing > 50 cells were counted and the experiments were performed in triplicate.

### Cell migration assay

A Transwell® migration assay was performed as previously reported [19]. In brief, hFOB.1.19 or U2OS cells (4 × 10^5^ cells/ml) were seeded in serum-free RPMI1640 or F12K medium in the top chamber of a Transwell® insert. The medium containing 20% FBS in the lower chamber served as a chemoattractant. After incubation for 24 h at 37 °C, the cells on the top side of the membrane were removed with a cotton swab, and those on the bottom side were fixed with methanol for 20 min and then stained with crystal violet (0.1% in PBS) for 15 min. Six randomly selected fields per well were photographed, and the numbers of migrated cells were counted.

### Scratch wound healing assay

A monolayer scratch wound assay was employed as previously described [20]. Briefly, cells (4 × 10^5^ cells/well) were seeded in 12 well plates and grown to nearly 100% confluence. A scratch wound was generated with a 200 μl pipette tip. Wound closure was photographed at 0 and 48 h.

### RNA interference (RNAi) assay

The p-EGFP-scramble, p-EGFP-green fluorescent protein (GFP), p-EGFP-CPXM2-RNAi, VSVG expression plasmid, virion-packaging elements (pHelper 1.0) and frozen glycerol bacterial stocks were purchased from Nanjing KeyGen Biotech Co, Ltd. (Nanjing, China). To achieve 70–80% confluence, 293 T cells (2 × 10^6^) were seeded and maintained in DMEM (10% FBS) at 37 °C for 24 h in 6-well dishes. A total of four plasmids, including 10 μg p-EGFP-CPXM2-RNAi or p-EGFP-scramble, 5 μg p-EGFP- GFP, 5 μg packaging vector pHelper 1.0 and 5 μg VSVG expression plasmid vector were added to Opti-MEM (cat. no. 31985062, Thermo Fisher Scientific, Inc.,) to a final volume of 1.0 ml. A total of 50 μl Lipofectamine® (cat. no. 11668027, Thermo Fisher Scientific, Inc.) was added to 950 μl FBS-free medium. The two components were mixed and added to the cells. Lentiviral particles were harvested 48 h post-transfection, and the virus titer was determined by counting GFP-expressing cells under a fluorescence microscope (magnification, × 200, Nikon Diaphot 300®) 36 h after transfection.

### Patients and tissue samples

Our research was performed with the approval of the Research Ethics Committee of First Hospital of Jilin University. The participants signed written informed consent prior this research. Tissues were gathered from 36 patients who underwent surgical resection at First Hospital of Jilin University between January 2006 and June 2013. The patients were chosen on account of the following principles: pathological diagnosis of OS; no prior or second tumor; no history of chemotherapy and radiotherapy. All excised tissues were immediately frozen in liquid nitrogen and stored at − 80 °C for the following study. The histologic grade and histologic subtypes were classified according to the 2013 WHO classification of bone tumors. The clinicopathologic parameters of osteosarcoma patients, containing age, gender, distant metastasis, histologic grade, histologic subtypes, Enneking clinical and TNM stage, were summarized and shown as Table 1. Histologically non-cancerous bone tissue was gained from 36 knee arthritis patients who was treated at The Second Hospital of Jilin University between January 2007 and October 2013, including 60 men and 36 women with an average age of 64 years.

**Table 1:** Expression of CPXM2 and clinicopathological characteristics in osteosarcoma patients

Item	N	CPXM2 positive	CPXM2 negative	*P*
Tumor tissue	36	21	15	< 0.001^*^
Noncancerous bone tissue	36	10	26	
Age (years)				
≤ 19	17	10	7	0.912^a^
> 19	19	11	8	
Sex				
Male	21	12	9	0.876^a^
Female	15	9	6	
Histologic grade				
I	4	1	3	
II	7	4	3	< 0.05^*^
III	25	16	9	
Histologic subtypes				
Osteoblastic	18	15	3	< 0.01^*^
Fibroblastic	4	1	3	
Chondroblastic	3	1	2	
Mixed	11	4	7	
Enneking clinical stages				
I-II	14	5	9	< 0.01^*^
III	22	16	6	
Response to chemotherapy				
Poor	9	5	4	0.926^a^
Good	7	4	3	
NA (*n* = 12)	20			
TNM stages				
I- II	14	5	9	< 0.01^*^
III-IV	22	16	6	
Pulmonary metastasis				
+	22	17	5	< 0.001^*^
-	14	4	10	
History of trauma or bone fracture				
+	11	7	4	0.756
-	25	14	11	
E-Cadherin				
+	12	4	8	< 0.01^*^
**-**	24	17	7	

^a^Statistical significance was determined with the χ^2^ test/χ^2^ Goodness-of-Fit Test

^*^Statistical significance was found with the Chi-square test/Chi-Square Goodness-of-Fit Test

### Immunohistochemistry

Sections were stained with an anti-CPXM2 polyclonal antibody using IHC in the Department of Pathology at our hospital. In brief, after deparaffinating, rehydrated in graded ethanol, antigen retrieval with Citrate Buffer pH 6.0 (1:300 dilution; ZLI-9065, ZSGB-Bio, Beijing, China) and blocking, slides were stained with a rabbit anti-CPXM2 polyclonal antibody (1:250 dilution; Absin Bioscience, Shanghai, China) at 4 °C overnight. Normal rat IgG (1:50 dilution; D110504, Sangon Biotech, Shanghai, China) instead of the primary antibody was used as a control. Subsequently, after washing with PBS, a horseradish peroxidase (HRP)-conjugated secondary antibody (1:2000; goat anti-rat cat. no. A0192, Beyotime, Jiangsu, China) was added and incubated at room temperature for 1 h. Then, these sections were stained by 3,39-diaminobenzidine (DAB) (GK500705, Gene Tech, Shanghai, China) and counterstained with hematoxylin. A modified H score system was used to semi-quantitate CPXM2 expression, as previously described [21]. Briefly, the maximal intensity of staining (0, negative; 1, weak; 2, moderate; and 3, strong) was multiplied by the percentage of positive tumor cells (0–100%) to generate the modified H score (range: 0–300). CPXM2 expression was categorized as high or low based on the median H score.

### Statistical analysis

Analyses were performed using Microsoft Excel 2010 (Microsoft, Redmond, WA, USA), GraphPad Prism7 (GraphPad, San Diego, CA, USA), and SPSS statistical software for Windows, version 22 (SPSS, Chicago, IL, USA). Paired or unpaired Student’s *t-*tests were performed for continuous variables. Pearson’s χ^2^ test and Fisher’s exact test were used for categorical comparisons. Survival analyses were conducted using the Kaplan-Meier method and differences in survival were examined using the log-rank test. Univariate and multivariate survival analyses were conducted using the Cox proportional hazards regression model. Statistical significance was defined as a value of *P* < 0.05. All statistical tests were two-sided.

## Results

### CPXM2 is overexpressed in osteosarcoma cell lines

**Fig. 1:**
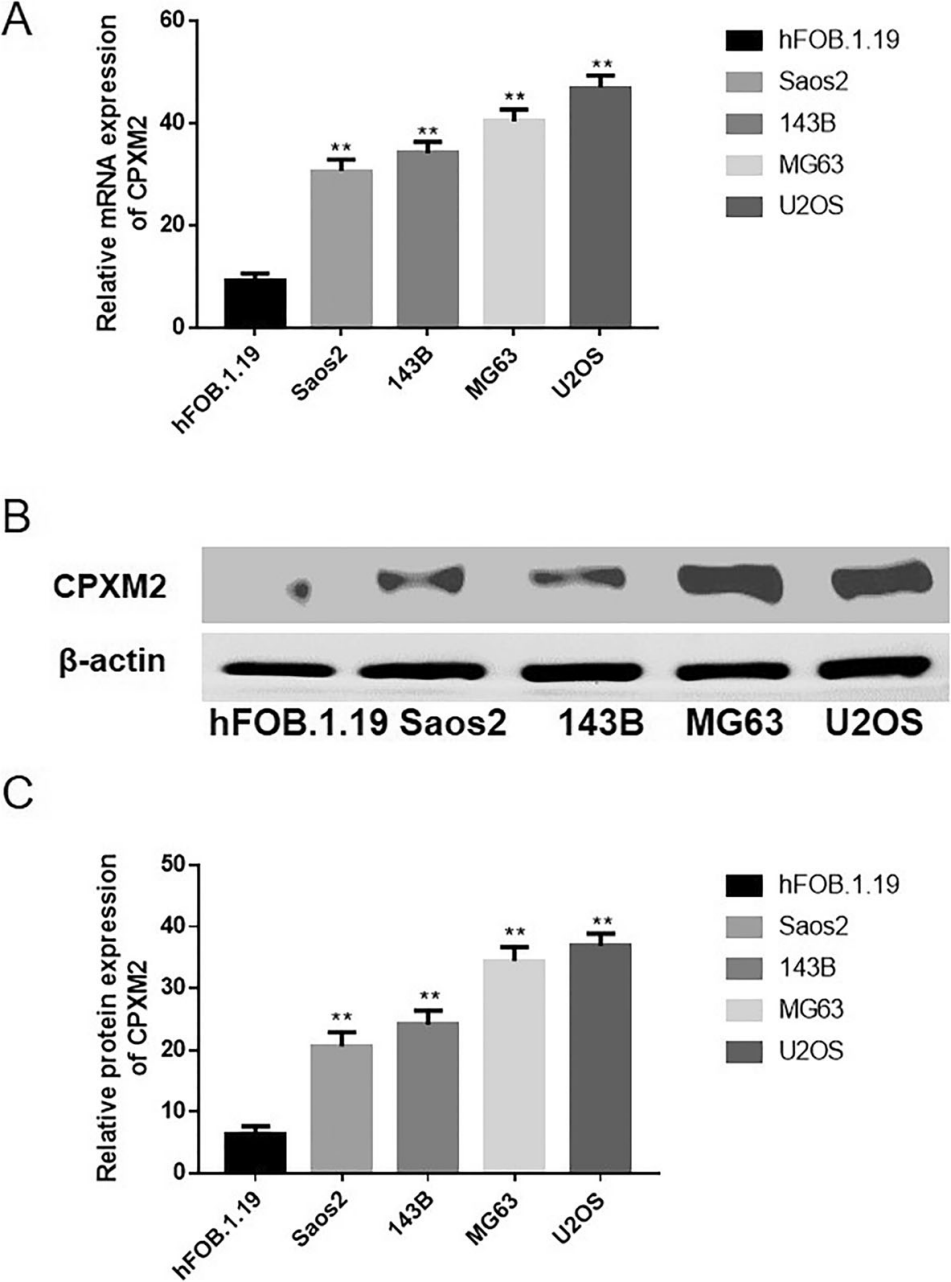
Expression of CPXM2 in osteosarcoma cell lines. **a** Relative mRNA expression level of CDN12 in osteosarcoma cell lines versus fetal osteoblast cells. **b** Protein expression of CPXM2 in and osteosarcoma cell lines versus fetal osteoblast cells. **c** The relative protein expression of CPXM2 in and osteosarcoma cell lines versus fetal osteoblast cells. ^**^P < 0.01 vs. fetal osteoblast cells. OS, osteosarcoma; CPXM2, Carboxypeptidase X, M14 family member 2

The expression of CPXM2 in osteosarcoma cell lines (Saos2, 143B, MG63, and U2OS) and a human fetal osteoblast cell line (hFOB.1.19) were examined via RT-qPCR and western blotting. It was determined that the CPXM2 were low expressed in hFOB.1.19 cells at both mRNA and protein levels, but high expressed in osteosarcoma cell lines Saos2, 143B, MG63, and U2OS (Fig. 1a-c).

### Expression of CPXM2 in human osteosarcoma tissues was upregulated and predicts an unfavorable prognosis

**Fig. 2:**
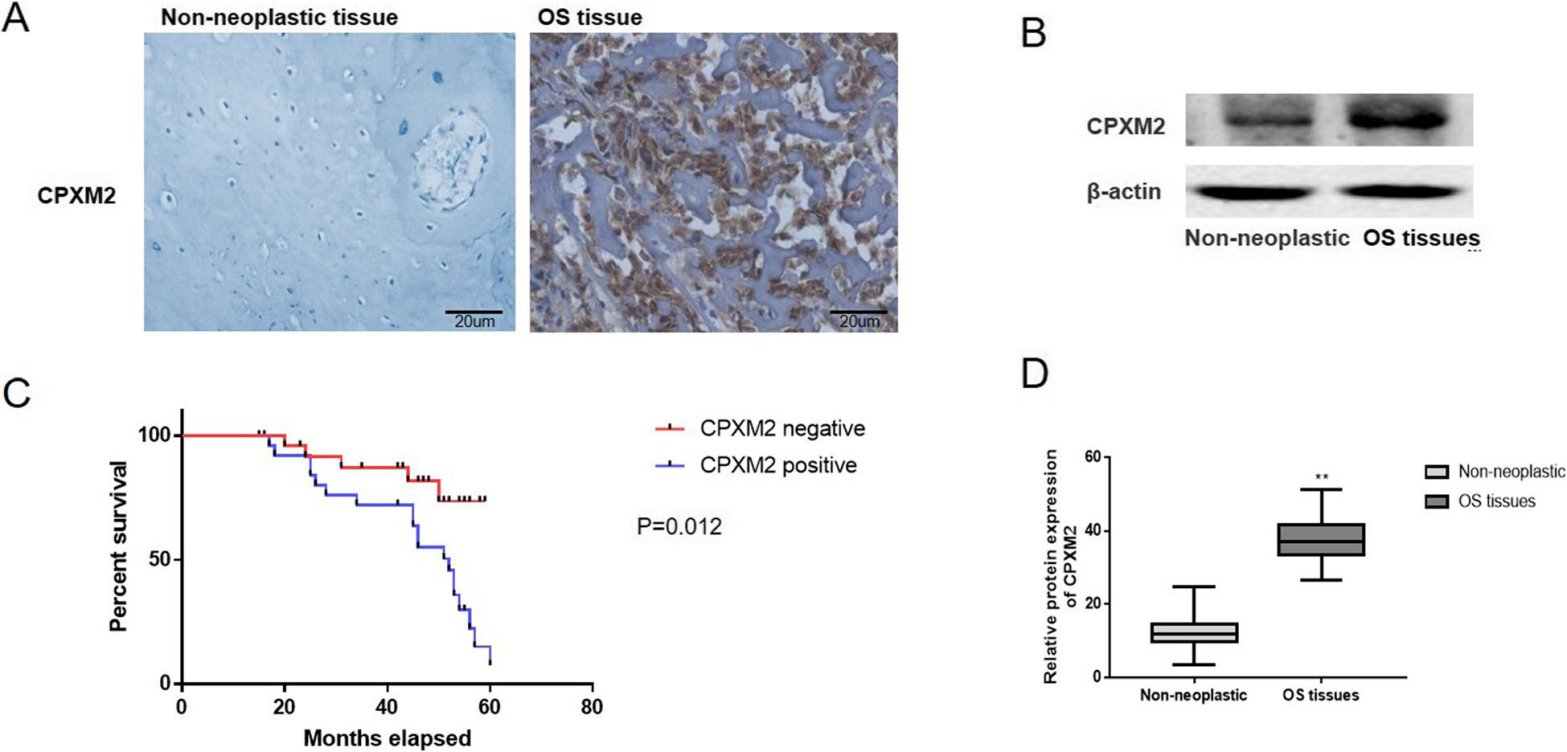
The expression of CPXM2 in osteosarcoma patients. **a** Microscopic analysis of CPXM2 protein expression in osteosarcoma tissues versus bone tissues. Scale bars, 20 μm. **b** Protein expression of CPXM2 in osteosarcoma tissues versus bone tissues. **c** Kaplan-Meier Survival Curves and the Log-Rank Test were used in analysis of association between CPXM2 and survival time. **d** The relative protein expression of CPXM2 in osteosarcoma tissues versus bone tissues. ^**^P < 0.01 vs. the bone tissues. OS, osteosarcoma; CPXM2, Carboxypeptidase X, M14 family member 2

Additionally, the CPXM2 expression was investigated in 36 osteosarcoma tissue samples and 36 noncancerous tissue samples. As it revealed in Fig. 2a, the CPXM2 is mainly expressed in the cytoplasm of the osteosarcoma cells. The cells that showed CPXM2 overexpression in noncancerous bone tissue were osteoblasts, and the tumor cells with CPXM2 expression derived from osteoblasts in tumor tissues. Also, CPXM2 was highly expressed in 58.33% (21/36) of osteosarcoma tissues and 27.78% (10/36) of non-neoplastic bone tissues (Table 1); The association between CPXM2 and clinical features was also evaluated, and CPXM2 expression was not meaningfully associated with the age (*P* = 0.912), sex (*P* = 0.876), response to chemotherapy (*P* = 0.426), and history of trauma or bone fracture (*P* = 0.756) in osteosarcoma patients. However, CPXM2 expression was meaningfully associated with Enneking clinical stages (*P* = 0.003), TNM stage (*P* = 0.002), pulmonary metastasis at vascular level (*P* = 0.003) and, E-Cadherin expression (*P* = 0.004) in osteosarcoma patients. Furthermore, our data observed that the CPXM2 expression is mainly expressed (15/27, 71.4%) in the osteoblastic osteosarcoma (*P* = 0.002), and notably associated with histologic grade (*P* = 0.011) (Table 1).

Western blotting analysis was also performed with the patient tissues in order to analyze the variations between noncancerous tissues and osteosarcoma tissues in CPXM2 expression. As illustrated in Fig. 2b and d, CPXM2 was notably upregulated in the 36 osteosarcoma tissues vs. the 36 noncancerous bone tissues (*P* = 0.0005).

The association between CPXM2 and survival time were analyzed using the Kaplan-Meier survival curves and the log-rank test. As illustrated in Fig. 2c, patients with high level of CPXM2 expression (median survival, 34.27 months) had a notably shorter survival time than those whose with low expression level of CPXM2 protein (median survival, 46.24 months; *P* = 0.0053).

### Impact of CPXM2 on the proliferation and metastasis of fetal osteoblast cells

**Fig. 3:**
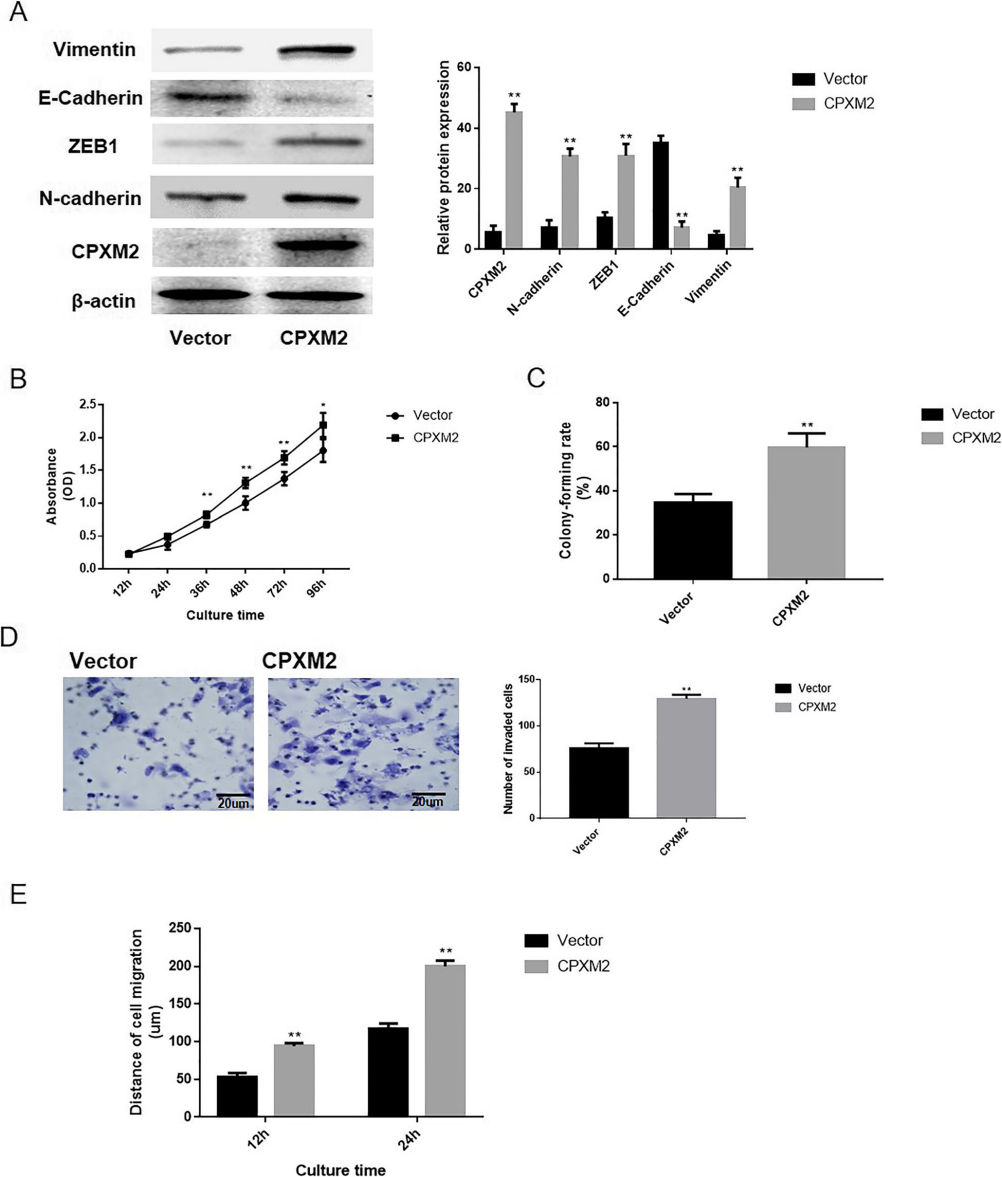
Impact of CPXM2 on the proliferation and metastasis of osteosarcoma cells in vitro. **a** Western blotting to examine the EMT process in the hFOB.1.19 cell line and the corresponding statistical analysis of EMT process components. **b** Growth curves of hFOB.1.19 cells as determined using the Cell Counting kit-8. **c** The abilities of hFOB.1.19 cells to form colonies under 2D culture condition were determined through colony formation assay. **d** Matrigel invasion assay to examine the invasive ability of hFOB.1.19 cells in vitro. Scale bars, 20 μm. **e** Wound-healing assay to detect the migration ability of hFOB.1.19 cells in vitro. ^**^P < 0.01 vs. the empty vector groups. CPXM2, Carboxypeptidase X, M14 family member 2; EMT, epithelial mesenchymal transition; OD, optical density

pNSE-IRES2-EGFP-CPXM2 and the empty vector pNSE-IRES2-EGFP (+) were transfected into hFOB.1.19 cells. Cells transfected with pNSE-IRES2-EGFP-CPXM2 (hFOB.1.19-CPXM2), and empty vector (hFOB.1.19 -Vector) were determined using G418 selection. As depicted in Fig. 3a, the protein expression level of CPXM2 in the hFOB.1.19-CPXM2 group was meaningfully higher versus the empty vector group (*P* = 0.0001). The EMT process in fetal osteoblast cells was also examined by western blotting and it is displayed that the expression levels of the mesenchymal markers N-cadherin (*P* = 0.0023) and Vimentin (*P* = 0.0001) and EMT related transcription factor ZEB1 (*P* = 0.0011) were meaningfully increased, while the epithelial marker E-cadherin (*P* = 0.0017) was decreased following CPXM2 overexpression.

To determine the influence of CPXM2 on the proliferative capacity of fetal osteoblast cells, the CCK-8 was used to explore the proliferative capacity of hFOB.1.19 cells. As depicted in Fig. 3b, there was a noteworthy alteration between the proliferation rates of the hFOB.1.19-CPXM2 and empty vector groups (*P* = 0.004). We also determined the ability of CPXM2-overexpressing cells to form colonies in 2D monolayer cultures (Fig. 3c). The number of colonies formed by CPXM2-overexpressing cells was significantly higher than the number formed by the Vector-transfected cells (*P* = 0.0002). Moreover, a Matrigel invasion assay was conducted to assess the metastatic capacity of fetal osteoblast cells; the observations revealed a meaningfully increased number of invasive cells (*P =* 0.0001) in the hFOB.1.19 -CPXM2 group, versus the empty vector group (Fig. 3d), indicating that CPXM2 promotes the migration ability of fetal osteoblast cells in vitro. Furthermore, wound-healing assay was conducted to explore the influence of CPXM2 on cell migration ability; it was displayed that after 12 and 24 h, the migration capacity of hFOB.1.19-CPXM2 cells was meaningfully enhanced, versus the empty vector group (*P =* 0.0013 and *P =* 0.0022, respectively; Fig. 3e). Taken together, these evidences indicated that that CPXM2 plays an active role in promoting osteosarcoma tumor aggressiveness via EMT modulation.

### Influence of CPXM2 knockdown on osteosarcoma cell metastasis

**Fig. 4:**
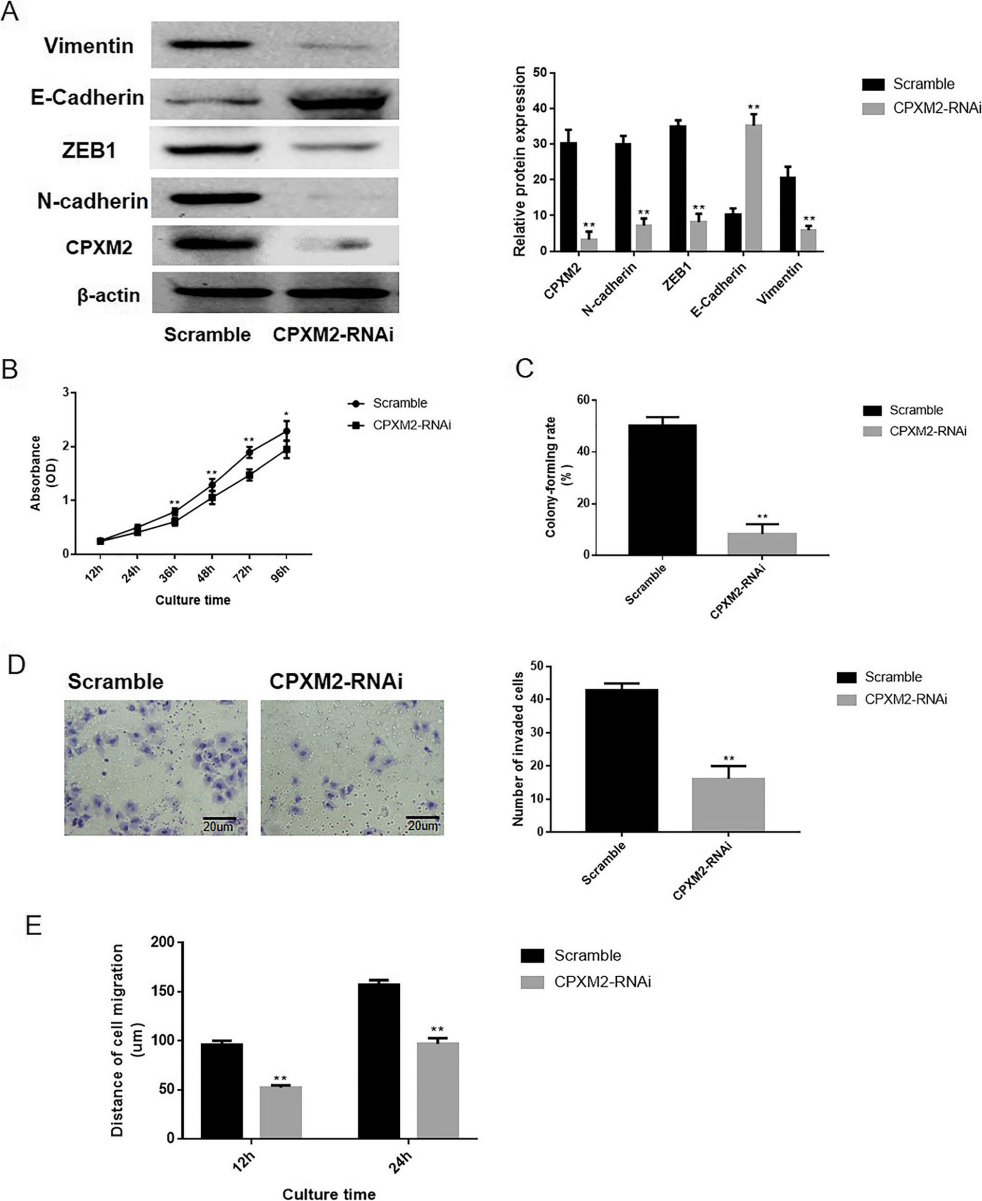
Loss of CPXM2 decreased the on the proliferation and migration ability of osteosarcoma U2OS cells. **a** Western blotting was used to examine the effects of silencing CPXM2 and EMT process in the osteosarcoma U2OS cells. **b** Growth curve of osteosarcoma cells detected by the CCK-8 assay. **c** The abilities of osteosarcoma cells to form colonies under 2D culture condition were determined through colony formation assay. **d** The Transwell chambers method was utilized to explore the impact of CPXM2 silence on the invasive ability of osteosarcoma U2OS cells in vitro. **e** The wound-healing assay was utilized to explore the migration ability of osteosarcoma cells in vitro. **P < 0.01, compared with the scramble group. CPXM2, Carboxypeptidase X, M14 family member 2; EMT, epithelial mesenchymal transition; OD, optical density

To explore the influence of CPXM2 on the metastatic ability of osteosarcoma cells, U2OS cells were transfected with p-EGFP-scramble or p-EGFP-CPXM2-RNAi plasmids. Western blotting was conducted to investigate the expression of CPXM2 and any alterations in the EMT process (Fig. 4a). The ratio of CPXM2 (*P* = 0.001), N-Cadherin (*P* = 0.0024), ZEB1 (*P* = 0.0026) and Vimentin (*P* = 0.0017) expression was significantly decreased while the expression of E-Cadherin (*P* = 0.0022) was significantly increased in U2OS cells (*P* = 0.0015) following CPXM2 silencing.

To determine the impact of CPXM2 on the malignant phenotype of osteosarcoma cell lines, the CCK8 method was used to draw the growth curve of the U2OS cell line. As depicted in Fig. 4b, the proliferation rate of CPXM2-RNAi transfected cells was significantly lower than that of the scramble-transfected cells (*P* = 0.0012). We also determined the ability of CPXM2-silenced cells to form colonies in 2D monolayer cultures (Fig. 4c). The number of colonies formed by CPXM2-silenced cells were significantly lower than the number formed by the scramble-transfected cells (*P* = 0.0030).

The results obtained from Matrigel invasion assay revealed that the amount of invasive U2OS cells in the CPXM2-silenced group was notably decreased (*P =* 0.0012; Fig. 4d), Moreover, Wound-healing assays (Fig. 4e) revealed that the migration distances of CPXM2-RNAi cells were significantly decreased after 12 and 24 h (*P =* 0.0017 and *P =* 0.0023, respectively) versus the scramble group.

## Discussion

Our knowledge about CPXM2 is still quite limited. Most studies have related CPXM2 to developmental diseases, mental disorders, and neurodegenerative diseases [11–14]. By analyzing differentially expressed genes in dermal fibroblasts from patients with Apert syndrome and controls, Cetinkaya et al. showed CPXM2 to be a gene with gene ontology terms associated with extracellular matrix organization, which may regulate early differentiation of connective tissues [11]. Using both growth-restricted and normal-term placentas, Sabri et al. recognized CPXM2 as one of the most upregulated genes in fetal growth restriction. Using bioinformatics analysis, these authors also suggested a potential connection between fetal growth restriction and gastrointestinal diseases [12]. There are at least 25 carboxypeptidases, several of which have already been identified as potential tumor biomarkers [4, 6, 22–24]. However, the underlying mechanism to explain the roles of these carboxypeptidases in modulating oncogenesis and tumor progression is still lacking. To date, a limited number of studies have surveyed the practical association between osteosarcoma carcinogenesis and CPXM2. Currently, we found that CPXM2 expression was overexpressed in osteosarcoma sections, and that the upregulation of CPXM2 promoted metastatic phenotype of fetal osteoblast cells. Furthermore, in the present study, we focused on the association between CPXM2 and 22 cases of pulmonary metastasis at the vascular level, our data revealed thatCPXM2 expression was significantly associated with distant metastasis in osteosarcoma patients. These observations suggested that abnormal expression of CPXM2 represented a potential target for genetic diagnosis, pathological staging, recurrence and prognosis in osteosarcoma. However, there are also 27.78% (10/36) of non-neoplastic bone tissues displayed CPXM2 expression. This result may be because CPXM2 is necessary for the proliferation of osteoblasts and is expressed in osteoblasts that are in the proliferative stage.

Currently, the specific impact of CPXM2 in osteosarcoma remains unclear. As EMT is a critical process that mediates tumor progression and metastasis, we postulate that CPXM2 may catalyze key molecules that regulate EMT in osteosarcoma. Our data represent that, CPXM2, an osteosarcoma proto-oncogene, significantly promoted the invasiveness of hFOB.1.19 cells. An initial investigation into the molecular mechanism of this effect was also performed, and it was determined that CPXM2 affects the EMT process via ZEB1, ultimately enhancing the metastatic capacity of fetal osteoblast cells. Moreover, an osteosarcoma cell line (U2OS) with a CPXM2-knockout was also settled up and it is illustrated that CPXM2-silencing lead to a suppression on metastasis in U2OS cells via modifying EMT process. To the best of our knowledge, this study is the first to investigate the prognostic value and molecular function of CPXM2 in cancers.

There are several unanswered questions in this study. First, we do not know the substrates of CPXM2. Second, further studies are needed to confirm whether CPXM2 can be used as a serum prognostic biomarker for osteosarcoma patients. Third, it is noted that the small sample size is a flaw of the current study, and that the character of CPXM2 as a gene biomarker may be investigated further by assessing the data of both inpatients and outpatients, and by collecting a larger number of samples.

## Conclusion

Currently, the specific impact of CPXM2 in osteosarcoma remains unclear. Our data represent that, CPXM2, as an osteosarcoma proto-oncogene, was found to accelerate tumor progression by promoting osteosarcoma cell migration via modulation of EMT process, indicating that a detailed study of this putative marker is warranted.

## Data Availability

The datasets used and/or analyzed during the present study are available from the corresponding author on reasonable request.

## Abbreviations

AJCC: American Joint Committee on Cancer
CPXM2: Carboxypeptidase X, M14 family member 2
EMT: epithelial mesenchymal transition
